# Impact of microencapsulated DL-methionine on intestinal health, immune gene expression, and economic returns in broilers chickens

**DOI:** 10.1016/j.psj.2026.106616

**Published:** 2026-02-10

**Authors:** Hossein Ali Ghasemi, Mohammad Ali Khazab, Seyed Abdullah Hosseini, Amir Meimandipour, Mahdi Ebrahimi

**Affiliations:** a Department of Animal Science, Faculty of Agriculture and Environment, Arak University, Arak, 38156-8-8349, Iran; b Department of Animal and Poultry Nutrition and Physiology, Animal Science Research Institute of Iran, Agricultural Research, Education and Extension Organization (AREEO), Karaj, Iran; c Department of Animal Biotechnology, National Institute of Genetic Engineering and Biotechnology (NIGEB), Tehran, Iran; d Research and Development Department, Zist Pishro Co Ltd, Tehran, P.O. Box 7133864343, Iran

**Keywords:** Microencapsulated DL-methionine, Broiler performance, Immune gene expression, Economic efficiency, Gut health

## Abstract

A 42-day study evaluated the efficacy of crystalline DL-methionine (**DL-Met**) at the recommended level (**control**) versus microencapsulated DL-methionine (**CM**) at 60%, 70%, 80%, 90%, and 100% of the commercial recommended levels (**CM60, CM70, CM80, CM90**, and **CM100**) in broiler chickens. A total of 1,260 one-day-old Arian broiler chicks were assigned to 6 experimental groups with 7 replicates of 30 chicks per group. The results showed that average daily gain (**ADG**) and the European performance index were significantly higher in the Control, CM80, CM90, and CM100 groups compared to CM60 during the 22–42-day period and over the entire 1–42-day period (*P* < 0.05). CM90 also outperformed the Control and CM70 in ADG (*P* < 0.05), and feed conversion ratio was significantly improved in CM80, CM90, and CM100 compared to the Control (*P* < 0.05). Ileal digestibility of dry matter and crude protein improved in CM80 and higher treatments, with AMEn also higher in CM80 to CM100 compared to CM60 (*P* < 0.05). In the ileum, CM90 and CM100 increased populations of anaerobic bacteria and *Lactobacillus*, while reducing *Coliform* populations (*P* < 0.05). Histomorphological indices, including villus height and surface area in the jejunum and villus height-to-crypt depth ratio in the ileum, were enhanced in intermediate-to-high CM treatments. Gene expression analysis revealed that NF-kB and IL-6 expression were significantly lower in CM90 and CM100 compared to other treatments, except CM80 (*P* < 0.05). IL-10 expression was higher in CM80, CM90, and CM100 compared to CM60 (*P* < 0.05), while no significant differences were observed for IL-1β and IFN-γ between all treatments (*P* > 0.05). Economically, CM60 had the lowest feed cost but resulted in reduced net profit and efficiency. In contrast, CM80 to CM100, particularly CM90, improved economic returns through higher income and reduced feed cost per unit of weight gain (*P* < 0.05). In conclusion, replacing crystalline methionine with microencapsulated methionine at 80–90% of the commercial recommendation improved growth performance, gut health, immune modulation, and economic returns.

## Introduction

Methionine is an essential sulfur-containing amino acid that plays a vital role in the nutrition and health of poultry. As the first limiting amino acid in corn-soybean meal-based broiler diets, methionine is crucial for protein synthesis, cellular metabolism, and immune system maintenance (Zhang et al., 2018; Hassanpour et al., 2024). Beyond its role in protein synthesis, methionine is a precursor to S-adenosylmethionine (**SAM**), a key compound involved in DNA methylation and cellular defense through the synthesis of glutathione, an antioxidant that helps mitigate oxidative stress (Ghasemi and Nari, 2023; Ács et al., 2025). Studies have also demonstrated that methionine supplementation can improve intestinal morphology, increasing villus height and the villus height-to-crypt depth (**VH:CD**) ratio in the ileum, both of which are indicators of enhanced nutrient absorption capacity (Lin et al., 2024; Zeitz et al., 2019). Furthermore, methionine influences immune function through modulation of the NF-κB pathway, a key regulator of immune and inflammatory responses (Cai et al., 2025). NF-κB is activated during stress or infection, triggering the expression of pro-inflammatory cytokines such as IL-6 and IL-1β, which are involved in the acute-phase response and immune defense (Dong et al., 2024). In poultry, excessive NF-κB activation can lead to chronic inflammation, impairing both growth and immune function (Biabani et al., 2024; Mohammadi et al., 2024). Importantly, methionine supplementation has been shown to reduce NF-κB activation, decreasing pro-inflammatory cytokine expression and promoting anti-inflammatory cytokines such as IL-10 (Khatlab et al., 2019; Gong et al., 2025).

Given its multifaceted functions in poultry growth and health, methionine supplementation is a common practice in poultry nutrition to optimize performance and support immune function (Baolin et al., 2021; de Aguiar et al., 2025). However, the bioavailability of methionine remains a challenge due to its rapid degradation under the acidic conditions of the stomach, which limits its absorption and efficacy (Gong et al., 2024). A major concern in poultry production is the high cost of methionine, which can constitute a significant portion of total feed expenses. Since feed costs account for approximately 70% of total poultry production expenses, optimizing methionine supplementation efficiency is essential to reducing overall costs while maintaining performance (Zampiga et al., 2021). Consequently, there is a pressing need for strategies that enhance methionine utilization without increasing costs, providing potential benefits for poultry producers.

Encapsulation technology offers a promising solution to enhance amino acid utilization in poultry diets. This technique involves encapsulating amino acids, such as crystalline lysine and DL-methionine, in a protective coating, which prevents premature degradation in the stomach and ensures their controlled, gradual release in the small intestine, where absorption is most efficient (Prandini et al., 2013; Sun et al., 2020a, b). By improving amino acid stability and bioavailability, encapsulation enhances its efficiency in poultry nutrition while reducing nitrogen excretion, contributing to a more environmentally sustainable production system (Sun et al., 2020a). Previous studies have demonstrated that encapsulation of supplemental amino acids can improve growth performance and feed conversion efficiency in broilers, without negatively impacting feed intake (Collins, 2021). Microencapsulation is a specific form of encapsulation that deals with much smaller particles and offers more precise control over release, making it particularly effective in applications where controlled delivery is crucial (Contreras-López et al., 2024). This technology optimizes nutrient utilization, helping mitigate the effects of high feed costs and promoting more sustainable poultry production (Liu et al., 2024).

Given the challenges associated with methionine bioavailability and its high supplementation cost, this study hypothesizes that microencapsulation of DL-methionine (**CM**) can enhance its efficiency by improving stability and enabling controlled release in the small intestine. This approach could optimize growth performance, gut health, and immune function, while potentially reducing the levels of supplemental methionine required. Additionally, by mitigating excessive activation of the NF-κB pathway—an important regulator of inflammation and immune responses—microencapsulation may help maintain balanced immune function, thereby reducing chronic inflammation that could impair growth. Therefore, the objective of this study was to evaluate the effects of varying levels of CM on growth performance, gut morphology, microbiota composition, immune gene expression, and economic efficiency in broiler chickens. By focusing on both immune modulation and performance outcomes, this research aims to provide a comprehensive understanding of how microencapsulated methionine can contribute to more sustainable and efficient poultry production.

## Materials and methods

### Birds and treatments

All experimental procedures were conducted in accordance with ethical guidelines and were approved by the Animal Ethics Committee of Arak University (Approval number 1402-d-13327), adhering to the ARRIVE guidelines 2.0 for animal research. A total of 1,260 one-day-old Arian broiler chicks (unsexed) were individually weighed at placement and then randomly allocated to 6 dietary treatments with 7 replicate pens per treatment (30 birds per pen), totaling 42 experimental units. Random allocation after weighing was used to balance initial body weight and to ensure an even distribution of sexes among treatments and replicate pens. The treatments consisted of: (1) Control (corn–soybean-based diet supplemented with crystalline DL-Met to meet the breeder-recommended digestible methionine requirement) and (2) five levels of microencapsulated DL-Met (**CM60, CM70, CM80, CM90**, and **CM100**). The CM levels were designed to provide 60%, 70%, 80%, 90%, and 100% of the recommended digestible Met requirement from the microencapsulated source, based on a 72% purity of the product. The diets were formulated on a corn–soybean meal basis in two phases: starter (days 1–21) and grower (days 22–42). All diets, except for the methionine source/level, were isocaloric and isonitrogenous, with balanced levels of metabolizable energy, crude protein, digestible amino acids (lysine and threonine), minerals, and vitamin–mineral premixes to ensure that the observed effects could be attributed to methionine level. The composition and chemical analysis of the ingredients for each phase are provided in Table 1 (1–21 days) and Table 2 (22–42 days). All diets were prepared in mash form, with uniform physical quality.

**Table 1:** Ingredients and nutrient composition of the experimental diets (days 1–21).

	Dietary Treatments^1^					
Ingredient (%, as‑fed)	Control	CM60	CM70	CM80	CM90	CM100
Corn	56.53	56.53	56.53	56.53	56.53	56.53
Soybean meal	34.92	34.92	34.92	34.92	34.92	34.92
Corn gluten meal	1.78	1.78	1.78	1.78	1.78	1.78
Soybean oil	1.50	1.50	1.50	1.50	1.50	1.50
Dicalcium phosphate	2.18	2.18	2.18	2.18	2.18	2.18
Calcium carbonate	1.22	1.22	1.22	1.22	1.22	1.22
Sodium chloride	0.23	0.23	0.23	0.23	0.23	0.23
Sodium bicarbonate	0.08	0.08	0.08	0.08	0.08	0.08
DL-methionine (98%)	0.31	—	—	—	—	—
Microencapsulated DL-methionine (72%)	—	0.25	0.30	0.34	0.39	0.43
L-lysine HCl (78.8%)	0.33	0.33	0.33	0.33	0.33	0.33
L-threonine (98.5%)	0.23	0.23	0.23	0.23	0.23	0.23
Vitamin premix^2^	0.25	0.25	0.25	0.25	0.25	0.25
Mineral premix^3^	0.25	0.25	0.25	0.25	0.25	0.25
Inert filler (silica sand)	0.19	0.25	0.20	0.16	0.11	0.07
Calculated nutrient composition						
Metabolizable energy (kcal/kg)	2,902	2,902	2,902	2,902	2,902	2,902
Crude protein (%)	22.1	22.1	22.1	22.1	22.1	22.1
Calcium (%)	1.04	1.04	1.04	1.04	1.04	1.04
Available phosphorus (%)	0.52	0.52	0.52	0.52	0.52	0.52
Sodium (%)	0.16	0.16	0.16	0.16	0.16	0.16
Digestible methionine (%)	0.62	0.49	0.53	0.56	0.59	0.62
Digestible Met + Cys (%)	0.93	0.80	0.84	0.87	0.90	0.93
Digestible lysine (%)	1.22	1.22	1.22	1.22	1.22	1.22
Digestible threonine (%)	0.85	0.85	0.85	0.85	0.85	0.85
Dietary electrolyte balance (mEq/kg)	250	250	250	250	250	250
Analyzed value, %						
Crude protein	21.5	21.5	21.6	21.6	21.5	21.6
Total methionine	0.66	0.52	0.55	0.59	0.62	0.63
Total Met + Cys	1.00	0.87	0.91	0.94	0.97	1.01

1 Microencapsulated DL‑methionine (CM) treatments; CM60–CM100 were formulated with microencapsulated DL‑methionine containing 72% active DL-methionine (w/w; remaining fraction is encapsulating matrix) at 60%, 70%, 80%, 90%, and 100% of the commercially recommended methionine level, respectively. The Control diet contained DL‑methionine (98%) at the commercially recommended level. Values are on an as‑fed basis unless otherwise indicated.

2 Vitamin premix provided per kg of diet: vitamin A, 11,000 IU; vitamin D_3_, 1,800 IU; vitamin E, 36 mg; vitamin K_3_, 5 mg; vitamin B_12_, 5 µg; thiamine, 1.6 mg; riboflavin, 1.53 mg; niacin, 7.5 mg; pyridoxine, 3 mg; biotin, 1.53 mg; folic acid, 0.03 mg; choline, 1 mg; pantothenic acid, 12.24 mg; ethoxyquin, 0.125 mg.

3 Mineral premix provided per kg of diet: iron, 250 mg; zinc sulfate, 84 mg; manganese sulfate, 160 mg; iodine, 1.6 mg; copper sulfate, 20 mg; selenium, 0.2 mg; cobalt, 0.4 mg.

**Table 2:** Ingredients and nutrient composition of the experimental diets (days 22–42).

	Dietary Treatments^1^					
Ingredients (%, as‑fed)	Control	CM60	CM70	CM80	CM90	CM100
Corn	64.82	64.82	64.82	64.82	64.82	64.82
Soybean meal	28.72	28.72	28.72	28.72	28.72	28.72
Corn gluten meal	—	—	—	—	—	—
Soybean oil	2.03	2.03	2.03	2.03	2.03	2.03
Dicalcium phosphate	1.65	1.65	1.65	1.65	1.65	1.65
Calcium carbonate	1.06	1.06	1.06	1.06	1.06	1.06
Sodium chloride	0.20	0.20	0.20	0.20	0.20	0.20
Sodium bicarbonate	0.13	0.13	0.13	0.13	0.13	0.13
DL-methionine (98%)	0.25	—	—	—	—	—
Microencapsulated DL-methionine (72%)	—	0.21	0.24	0.28	0.31	0.34
L-lysine HCl (78.8%)	0.28	0.28	0.28	0.28	0.28	0.28
L-threonine (98.5%)	0.21	0.21	0.21	0.21	0.21	0.21
Vitamin premix^2^	0.25	0.25	0.25	0.25	0.25	0.25
Mineral premix^3^	0.25	0.25	0.25	0.25	0.25	0.25
Inert filler (silica sand)	0.16	0.20	0.17	0.13	0.10	0.07
Calculated nutrient composition						
Metabolizable energy (kcal/kg)	3,017	3,017	3,017	3,017	3,017	3,017
Crude protein (%)	18.9	18.9	18.9	18.9	18.9	18.9
Calcium (%)	0.85	0.85	0.85	0.85	0.85	0.85
Available phosphorus (%)	0.42	0.42	0.42	0.42	0.42	0.42
Sodium (%)	0.16	0.16	0.16	0.16	0.16	0.16
Digestible methionine (%)	0.51	0.42	0.44	0.47	0.49	0.51
Digestible Met + Cys (%)	0.78	0.69	0.71	0.74	0.76	0.78
Digestible lysine (%)	1.04	1.04	1.04	1.04	1.04	1.04
Digestible threonine (%)	0.74	0.74	0.74	0.74	0.74	0.74
Dietary electrolyte balance (mEq/kg)	230	230	230	230	230	230
Analyzed value, %						
Crude protein	18.4	18.5	18.5	18.6	18.5	18.4
Total methionine	0.55	0.45	0.47	0.49	0.52	0.54
Total Met + Cys	0.85	0.76	0.78	0.80	0.83	0.86

1 Microencapsulated DL‑methionine (CM) treatments; CM60–CM100 were formulated with microencapsulated DL‑methionine containing 72% active DL-methionine (w/w; remaining fraction is encapsulating matrix) at 60%, 70%, 80%, 90%, and 100% of the commercially recommended methionine level, respectively. The Control diet contained DL‑methionine (98%) at the commercially recommended level. Values are on an as‑fed basis unless otherwise indicated.

2 Vitamin premix provided per kg of diet: vitamin A, 11,000 IU; vitamin D_3_, 1,800 IU; vitamin E, 36 mg; vitamin K_3_, 5 mg; vitamin B_12_, 5 µg; thiamine, 1.6 mg; riboflavin, 1.53 mg; niacin, 7.5 mg; pyridoxine, 3 mg; biotin, 1.53 mg; folic acid, 0.03 mg; choline, 1 mg; pantothenic acid, 12.24 mg; ethoxyquin, 0.125 mg.

3 Mineral premix provided per kg of diet: iron, 250 mg; zinc sulfate, 84 mg; manganese sulfate, 160 mg; iodine, 1.6 mg; copper sulfate, 20 mg; selenium, 0.2 mg; cobalt, 0.4 mg.

Forty-two experimental units, each measuring 4.5 m² (2.5 × 1.8 m), were used in this study. Experimental units were randomly assigned to treatments and clearly labeled. The house temperature was set to 33°C and relative humidity was maintained at 60% for 48 hours before chick arrival. Temperature was gradually reduced by approximately 2.5°C each week as the chicks aged, while humidity was controlled through floor spraying. Temperature was monitored using a digital thermometer and two mercury thermometers. Each experimental unit was equipped with one feeder and one waterer, and from day 7 onwards, a hanging bucket feeder and bell-type drinker were used. Fresh, clean water and feed were provided ad libitum. The lighting program consisted of 23 hours of light and 1 hour of darkness.

### Characteristics of DL-methionine and microencapsulation

The DL-methionine used in this study was a high-purity crystalline product sourced from Evonik (Essen, Germany), with a molecular formula of C₅H₁₁NO₂S and a molecular weight of 149.21 g/mol. The purity of the imported DL-methionine was greater than 98%, as confirmed by HPLC analysis. To enhance its stability and bioavailability, DL-methionine was microencapsulated by Soroush Sabzeh Alborz (Karaj, Iran) using a process that involves coating the methionine particles with a protective shell to maintain its stability at temperatures up to 80°C. After microencapsulation, the resulting product contained 72% DL-methionine on a weight basis (active ingredient), as verified by HPLC analysis. Diet formulations for the CM treatments were adjusted for this 72% active content to achieve the intended methionine supplementation levels. The encapsulated particles had an average size of 25 ± 6 µm, with uniform distribution, which facilitated better control of nutrient release and provided protection against environmental factors such as moisture and oxygen, improving both the physical and chemical stability of the supplement.

### Growth performance evaluation

Growth performance was assessed based on several key parameters, including average daily gain (**ADG**), average daily feed intake (**ADFI**), feed conversion ratio (**FCR**), mortality rate, and the European performance index (**EPI**). ADG was calculated by measuring the difference in body weight at the beginning and end of each growth phase (1–21 days, 22–42 days, and 1–42 days), then dividing the weight change by the number of days in each phase. ADFI was determined by recording the total feed consumption per pen on a daily basis, dividing this by the number of birds in each pen, and calculating the average for the same periods as ADG. FCR was determined by dividing the total feed intake by the total weight gain for each phase, providing a measure of feed efficiency. Overall mortality rate was recorded throughout the study and expressed as a percentage of the initial number of birds, with mortality also considered when correcting performance parameters. The EPI, which combines weight gain, feed intake, and mortality into a single index, was calculated using the formula: EPI = [Body weight (kg) × Livability (%) × 100] / [Age (d) × FCR].

### Digestibility assay

To determine apparent ileal nutrient digestibility, chromium oxide (3 g/kg) was added to the diet as a marker three days prior to collecting ileal contents (on day 39). At the end of the experiment (on day 42), 2 birds from each replicate were selected based on the average body weight of the pen and euthanized. The contents of their ileum were collected, and the ileal samples, along with feed samples, were sent to the laboratory for analysis. Dry matter, crude fat, crude protein, and gross energy of both feed and excreta samples were determined using standard methods as outlined by the AOAC (AOAC Int., 2005, Arlington, VA, USA).

Apparent ileal digestibility of nutrients (**AID**) was calculated using the following formula (Nari and Ghasemi, 2020):

$$AID=\left[1-\left(marker_{d}/marker_{id}\times nutrient_{id}/nutrient_{d}\right)\right]\times 100$$

Where nutrient _d_ and marker _d_ are the concentrations of nutrients and chromium oxide in the feed, and nutrient _id_ and marker _id_ are the concentrations of the same nutrients and chromium oxide in the ileal contents.

For the calculation of nitrogen-corrected apparent metabolizable energy (**AMEn**), excreta samples were used (*n* = 7 per treatment). AMEn was calculated using the following equation: (Azizollahi et al., 2024)

$$AMEn\left(kcal/kgofdiet\right)=GE_{d}-\left[\left(GE_{ex}\times IF\right)+8.22\times \left(N_{d}--N_{ex}\times IF\right)\right]$$

Where GE _d_ represents the gross energy of the diet (kcal/kg), GE _ex_ is the gross energy of the excreta (kcal/kg), IF is the correction factor for determining the indigestible fraction of the diet (marker _diet_/marker _id_), N_d_ is the nitrogen concentration in the diet, N_ex_ is the nitrogen concentration in the excreta, and 8.22 corresponds to the gross energy of uric acid (kcal/kg).

### Gut microbial populations

On day 42 of the experiment, 3 birds from each replicate were selected and euthanized. The ileal and cecal contents were collected and pooled within each replicate (*n* = 7 replicates per treatment). To isolate and quantify the gut microbiota, 1 g of fresh digestive content from each gut section was mixed in a sterile environment with an anaerobic dilution solution (**ADS**) at a 1:10 ratio under CO₂ conditions. Subsequent dilutions in ADS were made for counting anaerobic bacteria, while further dilutions in phosphate-buffered saline were prepared for counting aerobic bacteria. Dilutions were carried out in a stepwise manner with factors of 10, including further dilutions at 10^-5^, 10^-7^, and 10^-9^ for the ileal and cecal samples. A 0.1 mL sample from each dilution was plated in duplicate on the surface of the appropriate agar medium. Wilkins-Chalgren Anaerobic Agar (Oxoid, Hampshire, UK) was used for total anaerobic bacteria; MRS Agar (De Man, Rogosa, and Sharpe, Merk, Darmstadt, Germany) was used for *Lactobacillus*; BSM Agar (Bifidobacterium Selective Medium, Sigma-Aldrich, Darmstadt, Germany) for *Bifidobacteria*; MacConkey Agar (Merk, Darmstadt, Germany) for *Coliforms*; EMB Agar (Eosin Methylene Blue, Oxoid, Hampshire, UK) for *Escherichia coli*; and TSC Agar (Tryptose Sulfite Citrate, Oxoid, Hampshire, UK) for *Clostridia* (Nari et al., 2020). Plates for anaerobic bacteria, *Lactobacillus, Bifidobacteria*, and *Clostridia* were incubated in an anaerobic CO₂ incubator with 5% CO₂, while the other agar plates were incubated in an aerobic incubator. The incubation temperature was set at 37°C, and all media were incubated for 48 hours. Bacterial counts were expressed as colony-forming units (**CFU**) per gram of sample.

### Intestinal histomorphology

To assess the morphology of the small intestine, 2 birds with body weights close to the average of each replicate were selected at the end of the rearing period (day 42). Following euthanasia, segments from the mid-duodenum, jejunum, and ileum, each measuring 2 to 2.5 cm in length, were collected. The samples were rinsed with a buffered solution and immediately fixed in 10% neutral buffered formalin. Histological processing was performed following standard protocols, which included gradual dehydration, clearing, and embedding in paraffin. Transverse sections of 5 µm thickness were prepared using a microtome (Typ1400, Leitz, Wetzlar, Germany), mounted on slides, and stained with Hematoxylin-Eosin. Images were captured using a light microscope (Olympus CX31, Shinjuku, Tokyo, Japan), and quantitative analysis of the histological indices was conducted using the image analysis software QWinPlus v3.1.0 (Leica Cambridge Ltd.; UK) (Sajadi Hezaveh et al., 2020). For each bird, two sections were examined, and at least 10 villi and 10 crypts were measured per section. The primary parameters evaluated included villus height (**VH**), villus width (**VW**), and crypt depth (**CD**). The villus surface area (**VSA**) were calculated using the formula: VSA = VH × (VW/2) × 2π.

### Gene expression measurement

To evaluate the expression of immune and antioxidant genes, liver tissue samples were collected (*n* = 7 per replicate). Following euthanasia, a 2-3 cm section from the mid-region of the liver was excised, washed with cold phosphate-buffered saline, immediately frozen in liquid nitrogen, and stored at −80°C until RNA extraction. Total RNA was extracted using an RNA extraction kit (Pars-Tous, Mashhad, Iran) according to the manufacturer’s instructions. The purity and quantity of the RNA were assessed using a microplate spectrophotometer (BioTek, USA) at A260/A280, and the RNA was stored at −80°C until cDNA synthesis. To eliminate genomic DNA contamination, the RNA was treated with DNase I, and cDNA synthesis was performed using a cDNA synthesis kit (SinaClon, Tehran, Iran). A total of 25 ng of cDNA template was used in each reaction, which was carried out in the presence of SYBR Green qPCR Master Mix (containing ROX). Thermal cycling conditions were set according to the MIQE guidelines and standard procedures. The target genes included nuclear factor kappa B (**NF-κB**), interferon-gamma (**IFN-γ**), interleukin-6 (**IL-6**), interleukin-10 (**IL-10**), interleukin-1 beta (**IL-1β**), and the reference gene glyceraldehyde-3-phosphate dehydrogenase (**GAPDH**). Specific primers for each gene, including amplicon length and GenBank accession numbers, are listed in Table 3. Relative gene expression was quantified using the 2^-ΔΔCt^ method (Livak and Schmittgen, 2001), where the Ct value of the target gene was normalized to the Ct value of GAPDH. The ΔΔCt value was calculated relative to the control group, ensuring that gene expression was accurately compared across treatments.

**Table 3:** Primers used for each gene in PCR.

Gene	Primer sequence 5′−3′	Length (nt)	GenBank number
NF-kB	F: CCTGGCTGTTGTCGAATACCT	154	NM_001001472.3
	R: CACTTTGTTCACATCTGCCCC		
IFN-γ	F: TTCAGATGTAGCTGACGGTGG	139	NM_205149.2
	R: CGGCTTTGACTTGTCAGTGTT		
IL-6	F: TTCAGCAATGGCAACAGCAATG	156	NM_204628.2
	R: ATAGCAACAAGCGTCGTATTTCAAC		
IL-10	F: GCTCTCACACCGCCTTGC	216	NM_001004414.4
	R: ACTGCTTAACTGCTATCACTAACTCTC		
IL-1β	F: TCTTCTACCGCCTGGACAGC	145	XM_046931582.1
	R: TAGGTGGCGATGTTGACCTG		
GAPDH	F: CAGAACATCATCCCAGCGTCCAC	134	NM_204305.2
	R: CGGCAGGTCAGGTCAACAACAG		

^1^NF-kB, Nuclear factor kappa B; IL-1β, interleukin-1 beta; IFN-γ, interferon gamma; IL-6, interleukin-6; IL-10, interleukin-10; and GAPDH, glyceraldehyde-3-phosphate.

*F* = forward primer; *R* = reverse primer.

### Economic evaluation

To assess the economic efficiency of treatments containing microencapsulated DL-methionine (CM60–CM100) compared to the crystalline form (control), the cost of the diet for each phase (starter and grower) was obtained from the formulation office. This included the prices of crystalline methionine, microencapsulated methionine, and the cost of the microencapsulation process, which covered materials, operations, and packaging. The cost of feed consumed per bird in each phase was calculated by multiplying the diet price by the actual feed consumption during that phase. The sum of the costs from both phases provided the total feed cost. Production costs were divided into two components: total variable cost (**TVC**), which included feed consumption, health and treatment expenses, labor, and the cost of day-old chicks; and total fixed cost (**TFC**), which encompassed depreciation, building, and equipment costs (estimated according to standard reference procedures and allocated per bird). The sum of TVC and TFC provided the total cost (**TC**). Total revenue (**TR**) was calculated as the revenue from broiler sales (final live weight × market price per kilogram on day 42) plus revenue from bedding sales per bird. Net profit (**NP**) was then determined by subtracting TC from total revenue, as follows: NP = TR − TC (Amer et al., 2021).

Economic efficiency was further assessed using several common indicators, including the benefit-to-cost ratio (**BCR** = TR/TC), net profit per unit of total cost (**NP/TC**), feed cost per unit of body weight gain, and relative economic efficiency. The relative economic efficiency was calculated as:

Relative Economic Efficiency (tested group) = [(NP/TC) (tested group) ÷ (NP/TC) (control group)] × 100. (Mohammed et al., 2021)

### Statistical analysis

Data analysis was conducted using SAS statistical software (version 9.4; SAS Institute, Cary, NC, USA), employing the General Linear Model (GLM) procedure. Percentage data were assessed for normality using the Univariate Procedure of SAS. If normality was not met, the data were transformed to arcsine values prior to further analysis. Differences between means were evaluated using the LSMEANS option in SAS, with adjustments for Tukey's test at a significance level of *P* < 0.05. To examine the effects of varying methionine levels (linear or quadratic), the orthogonal contrast method was applied. Statistical significance was considered at *P* < 0.001 or *P* < 0.05, with trends noted for *P*-values between 0.05 and 0.10.

## Results and discussion

### Growth performance

Table 4 presents the growth performance of broiler chickens up to 42 days of age. ADG during the 1–21–day period was unaffected by dietary treatments. However, during days 22–42 and across the entire 1–42–day experimental period, the Control, CM80, CM90, and CM100 groups exhibited significantly higher ADG than the CM60 treatment (*P* < 0.05). In addition, CM90 yielded higher ADG than CM70 and the Control (*P* < 0.05). Moreover, dietary supplementation with CM resulted in both linear and quadratic effects on ADG over the 1–42–day period (*P* < 0.01). Dietary treatments did not significantly affect ADFI through 42 days of age (*P* > 0.05). However, the FCR from 1 to 21 days was significantly lower in CM90 that in the Control (*P* < 0.05). During days 22–42, all treatments, except CM70, had lower FCR than CM60 (*P* < 0.05). Across the entire experimental period, CM80, CM90, and CM100 exhibited a lower FCR than the Control (*P* < 0.05), with CM90 showing the lowest overall FCR (*P* < 0.05). Mortality throughout the entire period was not affected by dietary treatments (*P* > 0.05). The EPI from 0 to 42 days was significantly higher in birds receiving CM80, CM90, and CM100 relative to CM60 and CM70 (*P* < 0.05). Additionally, CM supplementation exhibited both linear and quadratic effects on EPI (*P* < 0.001), and CM90 achieved the highest EPI compared with the Control, CM60p, and CM70 groups.

**Table 4:** Growth performance indices in broiler chickens fed a basal diet with crystalline DL-methionine (control) or microencapsulated DL-methionine provided at 60, 70, 80, 90, or 100% of the commercially recommended methionine level (CM60–CM100).

	Experimental groups								Contrast^2^	
Item^1^	Control	CM60	CM70	CM80	CM90	CM100	SEM	*P*-value	L	Q
ADG, g/d										
1-21 d	36.25	35.30	36.19	36.31	36.94	37.04	0.453	0.117	0.004	0.507
22-42 d	82.42^bc^	77.04^d^	79.56^cd^	84.31^ab^	86.45^a^	84.45^ab^	0.819	<0.001	<0.001	<0.001
1-42 d	59.34^bc^	56.17^d^	57.88^cd^	60.31^ab^	61.69^a^	60.74^ab^	0.465	<0.001	<0.001	0.001
ADFI, g/d										
1-21 d	49.97	48.29	49.41	48.23	48.70	49.03	0.500	0.136	0.623	0.969
22-42 d	162.5	158.7	160.1	161.6	161.8	162.0	1.89	0.704	0.097	0.531
1-42 d	106.2	103.5	104.7	104.9	105.2	105.5	0.94	0.462	0.106	0.579
FCR										
1-21 d	1.38^a^	1.37^ab^	1.37^ab^	1.33^ab^	1.32^b^	1.32^ab^	0.013	0.005	0.002	0.304
22-42 d	1.97^bc^	2.06^a^	2.01^ab^	1.92^cd^	1.87^d^	1.92^cd^	0.016	<0.001	<0.001	<0.001
1-42 d	1.79^ab^	1.84^a^	1.81^a^	1.74^bc^	1.71^c^	1.74^bc^	0.013	<0.001	<0.001	<0.001
Mortality, %	5.19	8.29	6.44	5.60	5.32	6.81	1.251	0.503	0.311	0.133
EPI^3^	319.0^bc^	282.9^d^	302.5^cd^	330.9^ab^	346.2^a^	330.8^ab^	5.21	<0.001	<0.001	<0.001

^a-d^ Means within a column not sharing the same superscript are different at *P* < 0.05. Values are means of 7 pens per treatment combination with 30 broiler chickens for growth performance.

1 ADG, Average daily gain, ADFI, average daily feed intake, FCR, feed conversion ration; EPI, European performance index.

2 Orthogonal polynomial contrasts were used to test the linear and quadratic effects of increasing dietary concentrations of microencapsulated DL-methionine (CM).

In this study, the inferior ADG and FCR in CM60 and CM70 suggest that the lower levels of microencapsulated methionine were below the practical requirements. By contrast, CM80–CM100 restored performance, and CM90 provided the best overall balance, as reflected by improved FCR and EPI. Notably, performance responses appeared to plateau at around CM90, as increasing CM from 90% to 100% did not provide additional improvement in ADG, FCR, or EPI. This pattern is consistent with a requirement-based response: once the practical methionine requirements are met, extra methionine is increasingly catabolized rather than retained as body protein, reducing biological and economic efficiency (de Aguiar et al., 2025). Therefore, CM100 was not inferior in absolute performance, but it was less efficient than CM90 because it offered limited incremental benefit relative to the additional methionine input and cost. Consistent with our results, Barekatain and Kluenemann (2023) demonstrated that transitioning from a methionine-deficient diet (∼60% of specification) to adequate (100%) and oversupplemented (120%) levels significantly improved body weight gain and reduced FCR, with only small differences among DL-Met, l-Met, and 2‑hydroxy-4-(methylthio)butanoic acid (MHA-FA; methionine hydroxy analog, free acid). In layer chicks, Zhang et al. (2024) observed that increasing dietary methionine from 0.31% to 0.43–0.54% improved growth and related physiological indices, with a linear decrease in FCR as methionine increased. Under heat or environmental stress, adequate methionine is reported to support performance and physiological resilience compared with suboptimal methionine supply (Yodseranee and Bunchasak, 2012; Ghasemi et al., 2014).

The improvements in FCR and EPI observed with CM80–CM100, particularly CM90, align with evidence that encapsulated amino acid technologies can enhance methionine efficiency and maintain performance at lower inclusion levels. In broilers and laying hens, Sun et al. (2020a, 2020b) showed that encapsulated lysine + methionine supplied at 80% of conventional levels maintained growth or production and FCR, whereas supply at 60% impaired performance. Beyond terrestrial monogastrics, Guo et al. (2020) observed that supplementing low-fishmeal diets for Pacific white shrimp with microencapsulated methionine at 0.15–0.20% significantly increased final weight and weight gain rate compared with the unsupplemented basal diet, supporting the broader utility of protected methionine. The superior EPI observed for CM80–CM100 in our trial likely reflects not only improved amino acid balance but also better redox and gut health status, as methionine is central to antioxidant and immune defense. Studies in stressed or challenged broilers indicate that methionine supply can enhance antioxidant capacity and reduce inflammatory responses (Pang et al., 2023) as well as protect intestinal tissue (Khatlab et al., 2019). Likewise, Oretomiloye et al. (2025) demonstrated that a microencapsulated complex of biofactors and antioxidants improved early growth, FCR, villus morphology, and systemic antioxidant parameters in cold-stressed broilers. Taken together, these findings support the conclusion that CM90 improved FCR and EPI through enhanced methionine utilization, potentially accompanied by better antioxidant and gut function.

### Nutrient digestibility

Based on the ileal digestibility results (Table 5), the experimental treatments did not significantly affect the digestibility of crude fat and ash. However, the CM80 and CM90 treatments significantly increased the digestibility of dry matter compared with the Control and CM60 groups (*P* < 0.05). Additionally, all experimental treatments, except CM70, resulted in improved crude protein digestibility compared to the Control and CM60 groups (*P* < 0.05). Crude protein digestibility was also higher in CM80 than in CM70 (*P* < 0.05). The AMEn was higher in treatments with CM supplementation at 80% or more of the recommended methionine levels (CM80, CM90, and CM100) compared to CM60 (*P* < 0.05). Moreover, the CM90 treatment showed a significant increase in AMEn compared to the Control group (*P* < 0.05). Orthogonal comparisons revealed that the digestibility of dry matter and crude protein were affected linearly and quadratically by increasing levels of CM in the diet (*P* < 0.05). A linear relationship was also observed between the increasing dietary CM levels and AMEn (*P* < 0.05).

**Table 5:** Nutrient digestibility of broiler chickens fed a basal diet with crystalline DL-methionine (control) or microencapsulated DL-methionine provided at 60, 70, 80, 90, or 100% of the commercially recommended methionine level (CM60–CM100).

	Experimental groups								Contrast^2^	
Item^1^	Control	CM60	CM70	CM80	CM90	CM100	SEM	*P*-value	L	Q
DM, %	78.81^b^	78.60^b^	79.80^ab^	81.43^a^	81.69^a^	80.27^ab^	0.733	0.027	0.039	0.029
CP, %	69.83^c^	69.51^c^	71.20^bc^	73.62^a^	73.10^ab^	72.35^ab^	0.624	<0.001	0.001	0.004
CF, %	78.96	79.45	80.95	80.30	79.32	79.41	0.698	0.372	0.452	0.250
CA, %	49.64	51.63	50.61	50.96	51.06	50.53	0.733	0.542	0.433	0.785
AMEn, kcal/kg	2987^bc^	2979^c^	3003^abc^	3019^ab^	3025^a^	3020^ab^	11.3	0.039	0.011	0.141

^a-c^ Means within a column not sharing the same superscript are different at *P* < 0.05. Values are means of 7 pens per treatment combination with 3 broiler chickens.

1 DM, dry matter; CP, crude protein; CF, crude fat, CA, crude ash; AMEn, apparent metabolizable energy corrected for nitrogen retention.

2 Orthogonal polynomial contrasts were used to test the linear and quadratic effects of increasing dietary concentrations of microencapsulated DL-methionine (CM).

These results suggest that CM supplementation can improve nutrient digestibility and energy utilization in broiler diets. The most consistent responses were observed for dry matter and crude protein digestibility in CM80 and CM90, relative to the Control and CM60 groups. A plausible mechanism is that microencapsulation protects methionine from rapid dissolution and early loss, thereby enabling a more gradual release along the small intestine (Guo et al., 2020; King et al., 2021). This may improve the temporal and spatial alignment between amino acid availability and absorption, supporting higher apparent utilization of protein and energy (as reflected here by improved CP digestibility and AMEn at CM80–CM100). In addition, improved intestinal integrity (supported by our histomorphology and immune-related outcomes) can further enhance nutrient absorption efficiency. This aligns with previous studies showing that microencapsulation of supplements enhances nutrient absorption and digestibility. For example, Syed et al. (2021) demonstrated that microencapsulated supplements improved nutrient digestibility in broilers. Microencapsulation, particularly for amino acids, is an effective strategy to enhance nutrient stability and facilitate controlled release within the digestive system (Liu et al., 2024). By limiting early losses and improving delivery to the intestine, microencapsulation may improve utilization and reduce nitrogen excretion (Contreras-López et al., 2024). Evidence from other species also supports improved efficiency with protected methionine. In lactating dairy cows, encapsulated DL-methionine improved lactation performance by enhancing protein efficiency (King et al., 2021; Seleem et al., 2024). In the present study, the increased AMEn in CM80, CM90, and CM100 compared to CM60 further indicates better energy utilization. One proposed mechanism is improved nutrient stability and protection from enzymatic degradation (Arslan et al., 2022). For amino acids, gradual intestinal release may increase absorption efficiency, contributing to higher digestibility and supporting improved performance (Contreras-López et al., 2024; Liu et al., 2024).

### Intestinal microflora

The results for microbial populations in the ileum and cecum of broiler chickens are presented in Table 6. In the ileum, the CM90 and CM100 treatments exhibited significantly higher populations of total anaerobic bacteria compared with the Control, CM60, and CM70 treatments (*P* < 0.05). Additionally, the CM80 treatment increased total anaerobic bacteria compared with CM60 (*P* < 0.05). *Lactobacilli* populations were also higher in the CM90 and CM100 treatments compared with the Control and CM60 treatments (*P* < 0.05), while populations in the Control, CM70, and CM80 treatments were higher than those in CM60 (*P* < 0.05). The total coliform population was significantly lower in the CM80, CM90, and CM100 treatments compared with CM60 (*P* < 0.05). In contrast, populations of *Bifidobacteria, Escherichia coli*, and *Clostridium* species in the ileum, as well as the populations of all measured bacteria in the cecum, were not significantly affected by the experimental treatments (*P* > 0.05).

**Table 6:** Bacteria population (log_10_ CFU/g) in the ileum and cecum of broiler chickens fed a basal diet with crystalline DL-methionine (control) or microencapsulated DL-methionine provided at 60, 70, 80, 90, or 100% of the commercially recommended methionine level (CM60–CM100).

	Experimental groups											Contrast^1^	
Item	Control	CM60	CM70		CM80	CM90	CM100	SEM		*P*-value		L	Q
Ileum													
Total anaerobes	8.21^bc^	7.69^c^		8.17^bc^	8.76^ab^	9.06^a^	8.93^a^		0.217		0.001	<0.001	0.071
*Lactobacilli*	7.31^b^	6.54^c^	7.46^ab^		7.84^ab^	8.03^a^	7.91^a^	0.190		<0.001		<0.001	0.003
*Bifidobacteria*	7.11	6.80	7.21		7.27	7.67	7.42	0.270		0.347		0.063	0.350
*Coliforms*	7.01^ab^	7.61^a^	6.85^ab^		6.28^b^	6.07^b^	6.17^b^	0.338		0.025		0.003	0.122
*E. coli*	6.10	6.70	6.21		5.54	5.44	5.39	0.394		0.156		0.011	0.316
*Clostridia*	5.83	6.23	5.81		5.49	5.11	5.05	0.327		0.124		0.010	0.611
Cecum													
Total anaerobes	9.42	9.14	9.45		9.89	10.21	9.70	0.337		0.301		0.113	0.202
*Lactobacilli*	8.64	8.17	8.58		8.78	8.95	8.91	0.410		0.781		0.172	0.551
*Bifidobacteria*	8.58	8.34	8.66		8.71	8.95	9.10	0.277		0.469		0.062	0.885
*Coliforms*	8.62	8.84	8.68		8.53	8.20	8.24	0.372		0.799		0.199	0.889
*E. coli*	8.50	8.84	8.35		8.11	7.90	7.69	0.475		0.584		0.072	0.733
*Clostridia*	7.40	7.54	7.44		7.08	6.78	6.86	0.389		0.638		0.105	0.771

^a-c^ Means within a column not sharing the same superscript are different at *P* < 0.05. Values are means of 7 pens per treatment combination with 3 broiler chickens.

1 Orthogonal polynomial contrasts were used to test the linear and quadratic effects of increasing dietary concentrations of microencapsulated DL-methionine (CM).

The findings of the current study demonstrate that CM supplementation, particularly at 90% and 100% of the recommended level (CM90 and CM100), increased ileal total anaerobes and lactobacilli while reducing coliforms. These changes suggest a shift toward a more favorable microbial balance, which may support gut health and performance. Methionine, beyond its role in protein synthesis, contributes to intestinal mucosal integrity and immune regulation (Gong et al., 2023). For example, methionine can enhance tight-junction expression and reduce intestinal inflammation, thereby supporting microbial homeostasis (Kwak et al., 2024). Furthermore, regulating the gut microbiota by increasing *lactobacilli* populations and decreasing pathogenic bacteria, such as coliforms, enhances nutrient absorption efficiency and strengthens the immune system (Liu and Kim, 2023). Previous studies have also reported a direct correlation between dietary methionine levels and positive changes in gut microbiota, growth performance, and overall poultry health (Ma et al., 2021; Liu et al., 2022a). Consistent with these observations, methionine has also been linked to improved microbial balance and reduced oxidative stress (Ghorbani et al., 2025). The positive effects observed in this study from methionine supplementation are likely enhanced by the protective role of microencapsulation. Encapsulation can protect methionine from early degradation and enable gradual release, increasing its availability in the lower gut (Sun et al., 2020a, Sun et al., 2020b). This process ensures that methionine is more available to the ileal microbiota.

The lack of significant effects on cecal bacterial populations suggests that the impact of encapsulated methionine may be localized to specific sections of the gut. The ileum is a primary site for nutrient absorption and a key region for diet–microbiota interactions (Pan and Yu, 2013). The ileal environment differs from the cecum in physiology and pH (Kumar et al., 2019). With gradual release occurring more distally in the small intestine, CM may exert stronger effects in the ileum than in the cecum. In contrast, the cecal microbial composition, which is primarily composed of fermentative bacteria, is less responsive to nutritional changes and is more influenced by fermentable substrates and metabolites (da Silva et al., 2024). Localized immune activity may also be more pronounced in the ileum, potentially amplifying dietary effects (Duangnumsawang et al., 2023). Collectively, these findings suggest that microencapsulated DL-methionine primarily influences ileal microbiota, which may contribute to improved gut function and production efficiency.

### Intestinal histomorphology

The results related to intestinal mucosal morphology are presented in Table 7. No significant differences were observed in any of the morphological parameters in the duodenum. However, in the jejunum, VW and VSA were significantly higher in the CM80, CM90, and CM100 treatments compared with the CM60 and CM70 treatments (*P* < 0.05). In the ileum, the CM80 and CM90 treatments exhibited significantly higher VH compared with the Control and CM60 treatments (*P* < 0.05). Additionally, VH was higher in the CM70 and CM100 treatments compared with CM60 (*P* < 0.05). The VH:CD ratio was significantly increased in the CM90 treatment compared with the Control and CM60 treatments (*P* < 0.05). The CM70, CM80, and CM100 treatments also showed higher VH:CD ratios compared with CM60 (*P* < 0.05). The highest VSA was observed in the CM90 treatment, which showed a significant difference compared with the CM60 and CM70 treatments (*P* < 0.05). Furthermore, the CM80 and CM100 treatments exhibited higher VSA compared with CM60 (*P* < 0.05).

**Table 7:** Morphological quantitative parameters and the number of small intestinal goblet cells (GC) of broiler chickens fed a basal diet with crystalline DL-methionine (control) or microencapsulated DL-methionine provided at 60, 70, 80, 90, or 100% of the commercially recommended methionine level (CM60–CM100).

	Experimental groups								Contrast^1^	
Item	Control	CM60	CM70	CM80	CM90	CM100	SEM	*P*-value	L	Q
Duodenum										
VH (µm)	1585	1526	1557	1630	1661	1594	37.1	0.151	0.059	0.111
VW (µm)	168.8	161.7	165.6	171.2	177.3	165.4	6.499	0.628	0.374	0.230
CD (µm)	208.8	214.7	210.9	201.8	197.6	211.7	9.701	0.810	0.466	0.202
VH:CD	7.81	7.30	7.44	8.10	8.49	7.55	0.404	0.313	0.203	0.096
VSA^2^ (mm)	0.841	0.777	0.811	0.876	0.923	0.828	0.0388	0.151	0.097	0.074
Jejunum										
VH (µm)	1194	1141	1182	1266	1286	1236	38.9	0.102	0.026	0.108
VW (µm)	208.0^ab^	194.7^b^	197.6^b^	228.4^a^	224.4^a^	222.8^a^	8.031	0.015	0.002	0.143
CD (µm)	211.8	217.3	207.8	204.8	211.0	217.4	6.030	0.628	0.862	0.087
VH:CD	5.67	5.26	5.70	6.23	6.10	5.75	0.258	0.144	0.101	0.028
VSA^2^ (mm)	0.780^ab^	0.700^b^	0.734^b^	0.906^a^	0.909^a^	0.864^a^	0.0419	0.002	0.001	0.048
Ileum										
VH (µm)	964^bc^	901^c^	1000^ab^	1074^a^	1084^a^	1039^ab^	29.7	0.001	<0.001	0.002
VW (µm)	177.7	168.0	166.2	176.0	181.1	179.6	6.818	0.540	0.036	0.842
CD (µm)	219.6	230.2	220.0	227.2	210.5	217.9	9.859	0.769	0.307	0.769
VH:CD	4.43^bc^	3.97^c^	4.65^ab^	4.78^ab^	5.20^a^	4.77^ab^	0.221	0.012	0.003	0.020
VSA^2^ (mm)	0.540^abc^	0.477^c^	0.522^bc^	0.594^ab^	0.616^a^	0.585^ab^	0.0277	0.010	<0.001	0.026

^a-c^ Means within a column not sharing the same superscript are different at *P* < 0.05. Values are means of 7 pens per treatment combination with 3 broiler chickens.

1 Orthogonal polynomial contrasts were used to test the linear and quadratic effects of increasing dietary concentrations of microencapsulated DL-methionine (CM).

2 VH, villus height; VW, Villus width; CD, Crypt depth; and VSA, Villus surface area (mm^2^) = 2π × (villus width /2) × villus height.

The histomorphological results of this study suggest that treatments containing microencapsulated methionine in the intermediate range (CM80–CM90) improved key intestinal absorption indices. In the jejunum, VW and VSA increased, while in the ileum, VH and the VH:CD ratio improved. No comparable effects were detected in the duodenum, indicating a site-specific response. This pattern aligns with the performance data, where CM80–CM90 produced the best ADG and FCR. Higher VH, VSA, and VH:CD generally indicate greater absorptive capacity, which may help explain the improved growth performance (Zeitz et al., 2019; Nari et al., 2020). Consistent with this interpretation, Miao et al. (2021) reported that increasing methionine, particularly under stress, improved VH and VH:CD. Changes in methionine source may also strengthen the epithelial barrier. For example, methionine hydroxy analogue has been shown to upregulate tight-junction proteins (claudin-1 and ZO-1), increase *Lactobacillus*, and enhance short-chain fatty acid concentrations, supporting mucosal health (Gong et al., 2023). Mechanistically, microencapsulation of amino acids can improve bioavailability by delivering nutrients more gradually, thereby optimizing absorption and utilization (Liu et al., 2024). In one study, encapsulated methionine and lysine increased jejunal VH and upregulated amino acid transporters (e.g., B0AT), supporting epithelial repair and junction integrity (Sun et al., 2020a). Therefore, the intermediate CM dose (CM80–CM90) may have maximized intestinal development and function without requiring higher total methionine input.

### Gene expression related to inflammatory and immune responses

**Figure 1:**
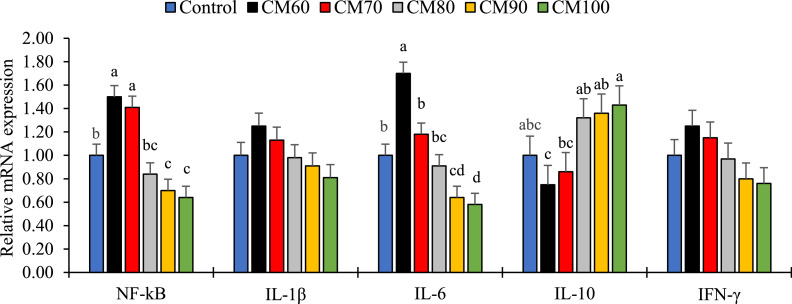
Relative mRNA expression of immune genes in liver of broiler chickens fed a basal diet with crystalline DL-methionine (control) or microencapsulated DL-methionine at 60, 70, 80, 90, or 100% of the commercially recommended methionine level (CM60–CM100). Bars show treatment means ± SEM (*n* = 7 pens per treatment; RNA extracted from pooled tissue of 3 birds per pen; normalized to glyceraldehyde-3-phosphate (GAPDH); expression calculated by the 2^−ΔΔCt^ method with the control set to 1.0). Different letters above bars indicate significant differences among treatments within each gene (one-way ANOVA followed by Tukey post-hoc test, *P* < 0.05). Gene abbreviations: NF-kB, nuclear factor kappa B; IL-1β, interleukin-1 beta; IFN-γ, interferon gamma; IL-6, interleukin-6; and IL-10, interleukin-10.

The results related to gene expression of NF-kB and specific genes within the NF-kB pathway are presented in Figure 1. Gene expression of NF-kB and IL-6 was significantly lower in the CM90 and CM100 treatments compared with all other treatments, except for CM80 (*P* < 0.05). A reduction in NF-kB gene expression was also observed in the CM80 and Control treatments compared with the CM60 and CM70 treatments (*P* < 0.05). IL-6 gene expression was lower in the CM80, CM70, and Control treatments compared with the CM60 treatment (*P* < 0.05). In contrast, IL-10 gene expression was significantly higher in the CM80, CM90, and CM100 treatments compared with CM60 (*P* < 0.05). No significant differences were observed among experimental treatments regarding the gene expression of IL-1β and IFN-γ (*P* > 0.05).

The findings of this study suggest that the reduction in NF-kB and IL-6 gene expression, alongside the increase in IL-10 in treatments with higher microencapsulated methionine (CM80–CM100), reflects a lower intestinal inflammatory tone and a shift toward anti-inflammatory signaling. This response may be related to improved methionine availability with microencapsulation, which can reduce inflammatory load at the gut mucosa. This pattern is consistent with evidence that adequate methionine supply (or functional derivatives such as methionine hydroxy analogue) can limit NF-kB activation by improving antioxidant status and inhibiting the TLR/MyD88/NF-kB pathway (Vijayan et al., 2014), thereby reducing the expression of pro-inflammatory cytokines like IL-6. Furthermore, methionine enhances the production of SAM, boosting methylation capacity, which can suppress the expression of inflammatory genes (Liu and Kim, 2023). In contrast, methionine deficiency disrupts the balance of NF-kB/IκBα and reduces SAM levels, leading to increased expression of NF-kB and IL-6 (Liu et al., 2022b; Mou et al., 2024). Similar responses have been reported in aquatic species, where higher dietary methionine reduces mRNA expression of NF-kBp65 and pro-inflammatory cytokines while upregulating anti-inflammatory cytokines, including IL-4/13B, IL-10, and IL-11 (Liang et al., 2022). Overall, microencapsulation may have provided a more consistent methionine supply to the intestinal tissue, supporting lower NF-kB/IL-6 and higher IL-10, which aligns with the improved growth performance observed.

### Economic efficiency

The results presented in Table 8 show that the feed cost per bird during the starter period was lowest for the CM60 and CM80 treatments (*P* < 0.05), although these values did not differ from those of the other treatments (*P* > 0.05), except for CM90 (*P* > 0.05). During the finishing period, the cost of the final diet and the average total diet cost for the entire experimental period, as well as the total cost per bird (including feed cost and additional costs), were lowest for the CM60 treatment, which was significantly different from all other treatments (*P* < 0.05). Furthermore, the CM70 and CM80 treatments exhibited lower feed costs for the final diet, total diet cost, and TC per bird compared to the Control and CM100 treatments (*P* < 0.05). The sale price of each bird at the end of the experimental period, based on weight gain and total income (from chicken sales and manure), was highest in the CM90 treatment, with a significant difference compared to all other treatments except for CM100 (*P* < 0.05). The CM100, CM80, and Control treatments also showed significantly higher sales prices per bird and TR compared to the CM70 and CM60 treatments (*P* < 0.05). In contrast, the CM60 treatment had the lowest bird sales and TR, which were significantly lower than all other treatments (*P* < 0.05). The NP, the BCR, NP/TC ratio, and relative efficiency were highest in the CM80, CM90, and CM100 treatments compared to the other experimental treatments (*P* < 0.05). Additionally, NP, BCR, NP/TC ratio, and relative efficiency were higher in the Control treatment compared to CM60 (*P* < 0.05), though similar to CM70 (*P* > 0.05). The feed cost-to-weight gain ratio was lowest in the CM90 and CM80 treatments, with a significant difference compared with the Control, CM60, and CM70 treatments (*P* < 0.05).

**Table 8:** Different economic efficiency measures of broiler chickens fed a basal diet with crystalline DL-methionine (control) or microencapsulated DL-methionine provided at 60, 70, 80, 90, or 100% of the commercially recommended methionine level (CM60–CM100).

	Experimental groups								Contrast^1^	
Item	Control	CM60	CM70	CM80	CM90	CM100	SEM	*P*-value	L	Q
Starter feed cost/ton, USD	241.4	238.2	239.3	240.4	241.6	242.8	-	-	-	-
Starter feed cost/bird, USD	0.253^a^	0.242^c^	0.250^ab^	0.241^c^	0.245^bc^	0.250^ab^	0.0018	<0.001	<0.001	<0.001
Grower feed cost/ton, USD	226.9	224.0	224.9	226.0	227.1	228.3	-	-	-	-
Grower feed cost/bird, USD	0.774^a^	0.743^d^	0.754^c^	0.762^bc^	0.771^ab^	0.777^a^	0.0026	<0.001	<0.001	0.334
Feed cost/bird, USD	1.027^a^	0.985^c^	1.004^b^	1.003^b^	1.016^ab^	1.027^a^	0.0037	<0.001	<0.001	0.870
Over feed cost/bird, USD	0.722	0.722	0.722	0.722	0.722	0.722	-	-	-	-
Total cost (TC), USD	1.749^a^	1.707^c^	1.726^b^	1.725^b^	1.738^ab^	1.749^a^	0.0037	<0.001	<0.001	0.870
Bird price/kg, USD	1.025	1.025	1.025	1.025	1.025	1.025	-	-	-	-
Value of bird at sale, USD	2.607^c^	2.459^e^	2.533^d^	2.639^bc^	2.696^a^	2.668^ab^	0.0115	<0.001	<0.001	<0.001
Return form litter, USD	0.031	0.031	0.031	0.031	0.031	0.031	-	-	-	-
Total return (TR)/bird, USD	2.638^c^	2.490^e^	2.564^d^	2.670^bc^	2.727^a^	2.699^ab^	0.0115	<0.001	<0.001	<0.001
Net profit/bird, USD	0.889^b^	0.783^c^	0.838^b^	0.945^a^	0.988^a^	0.949^a^	0.0121	<0.001	<0.001	<0.001
BCR^2^	1.490^b^	1.441^c^	1.467^bc^	1.530^a^	1.551^a^	1.525^a^	0.0074	<0.001	<0.001	<0.001
Net profit/TC	0.508^b^	0.459^c^	0.485^bc^	0.548^a^	0.569^a^	0.543^a^	0.0074	<0.001	<0.001	<0.001
Relative efficiency	100.0^b^	90.1^c^	95.3^bc^	107.5^a^	111.6^a^	106.6^a^	1.34	<0.001	<0.001	<0.001
Feed Cost/weight gain, USD	0.412^a^	0.417^a^	0.413^a^	0.396^bc^	0.393^c^	0.403^b^	0.0021	<0.001	<0.001	<0.001

^a-c^ Means within a column not sharing the same superscript are different at *P* < 0.05. Values are means of 7 pens per treatment combination with 30 broiler chickens per replicate pen.

1 Orthogonal polynomial contrasts were used to test the linear and quadratic effects of increasing dietary concentrations of microencapsulated DL-methionine (CM).

2 BCR (benefit–cost ratio = TR/TC).

Given that CM represents microencapsulated methionine, the price of the diet fluctuates with changes in methionine levels and the cost of the microencapsulation process. Therefore, the economic assessment should prioritize net profit rather than feed cost alone. Although CM60 had the lowest feed costs, it also produced the lowest sales value and total return, resulting in the lowest net profit (0.783 USD), benefit-to-cost ratio (1.441), net profit-to-total cost ratio (0.459), and relative efficiency (90.1%). In contrast, despite higher feed costs with increasing CM inclusion (e.g., starter feed increased from 238.2 to 242.8 USD/ton and grower feed from 224.0 to 228.3 USD/ton), the CM80–CM100 treatments improved economic returns. Notably, CM90 achieved the highest total income (2.727 USD) and sales per bird (2.696 USD), along with the lowest feed cost per unit of weight gain (0.393). CM90 also had the highest net profit (0.988 USD), benefit-to-cost ratio (1.551), net profit-to-total cost ratio (0.569), and relative efficiency (111.6%). This pattern aligns with the principle that the optimal supplementation level occurs when marginal income from added performance equals the marginal cost of supplementation (Vedenov and Pesti, 2010). Accordingly, higher amino acid inclusion can increase profitability if it improves ADG and FCR enough to reduce cost per unit of gain. Similar conclusions have been reported for threonine (Abo Ghanima et al., 2023) and for lysine and methionine, where moderate increases above reference levels produced the highest net income (Bouyeh, 2013). Recent work also indicates that higher lysine profiles can improve economic efficiency despite higher feed costs, due to better ADG and FCR (Mohamed et al., 2025). Overall, even accounting for microencapsulation costs, the most favorable economic response in this study occurred at intermediate CM levels, particularly CM80–CM90, rather than at the lowest-cost diet (CM60).

A final consideration is that responses to methionine typically follow a requirement-type curve, where benefits increase up to the point of adequacy and then plateau. Accordingly, supplying methionine beyond the optimal range may become less efficient because additional methionine is more likely to be catabolized rather than retained as body protein and may provide limited incremental improvements in performance relative to added input (Zamani et al., 2021; de Aguiar et al., 2025). This biological ‘diminishing returns’ effect offers a rationale for why CM100 did not provide clear advantages over CM90 in the present study.

## Conclusions

This study demonstrates that microencapsulated synthetic DL-methionine can reduce crystalline methionine dosage by up to 30% without compromising performance, particularly when the replacement level is within the CM80–CM90 range. In contrast, the CM60 diet, despite meeting other amino acid requirements, was insufficient for optimal performance. Microencapsulation improved gut health, including enhanced intestinal histomorphology, increased villus height and absorptive surface area, and a higher VH:CD ratio, along with better dry matter and crude protein digestibility and higher AMEn in the CM80-CM100 treatments. The CM90–CM100 treatments also promoted a beneficial shift in ileal microbiota, with increased anaerobic bacteria and *Lactobacillus* populations and reduced coliforms. Gene expression analysis indicated reduced NF-κB and IL-6 and increased IL-10, supporting anti-inflammatory effects. While CM60 had the lowest feed cost, it resulted in lower income and profitability. In contrast, CM80–CM100, especially CM90, improved economic outcomes, with higher income and reduced feed cost per unit of weight gain. Based on these results, replacing crystalline methionine with microencapsulated methionine in the 80–90% range is recommended for optimizing gut health, nutritional efficiency, and profitability in similar production systems, with the optimal level determined by cost and pricing factors.

## CRediT authorship contribution statement

**Hossein Ali Ghasemi:** Writing – review & editing, Writing – original draft, Software, Methodology, Formal analysis, Conceptualization. **Mohammad Ali Khazab:** Writing – original draft, Visualization, Software, Investigation, Data curation. **Seyed Abdullah Hosseini:** Writing – review & editing, Validation, Project administration, Methodology. **Amir Meimandipour:** Writing – review & editing, Software, Resources, Methodology. **Mahdi Ebrahimi:** Writing – review & editing, Methodology, Investigation, Conceptualization.

## Disclosures

The authors declare that the study was carried out without any financial or commercial relationships that could be construed as a potential source of conflict of interest.
